# Identifying Pathogen and Allele Type Simultaneously in a Single Well Using Droplet Digital PCR

**DOI:** 10.1128/msphere.00493-22

**Published:** 2023-01-10

**Authors:** Kosuke Notsu, Hala El Daous, Shuya Mitoma, Xinyue Wu, Junzo Norimine, Satoshi Sekiguchi

**Affiliations:** a Graduate School of Medicine and Veterinary Medicine, University of Miyazaki, Miyazaki, Japan; b Faculty of Veterinary Medicine, Benha University, Toukh, Egypt; c Department of infectious disease, Faculty of Medicine, University of Miyazaki, Miyazaki, Japan; d Department of Veterinary Science, Faculty of Agriculture, University of Miyazaki, Miyazaki, Japan; e Center for Animal Disease Control, University of Miyazaki, Miyazaki, Japan; University of Maryland School of Medicine

**Keywords:** diagnostic, digital PCR, bovine leukemia virus, enzootic bovine leukosis, viral load quantification, major histocompatibility complex, allele-specific PCR, elite controller, superspreader, BoLA-DRB3*009:02, BoLA-DRB3*016:01

## Abstract

In the transmission control of chronic and untreatable livestock diseases such as bovine leukemia virus (BLV) infection, the removal of viral superspreaders is a fundamental approach. On the other hand, selective breeding of cattle with BLV-resistant capacity is also critical for reducing the viral damage to productivity by keeping infected cattle. To provide a way of measuring BLV proviral load (PVL) and identifying susceptible/resistant cattle simply and rapidly, we developed a fourplex droplet digital PCR method targeting the BLV *pol* gene, BLV-susceptible bovine major histocompatibility complex (*BoLA*)-DRB3*016:01 allele, resistant DRB3*009:02 allele, and housekeeping RPP30 gene (IPATS-BLV). IPATS-BLV successfully measured the percentage of BLV-infected cells and determined allele types precisely. Furthermore, it discriminated homozygous from heterozygous carriers. Using this method to determine the impact of carrying these alleles on the BLV PVL, we found DRB3*009:02-carrying cattle could suppress the PVL to a low or undetectable level, even with the presence of a susceptible heterozygous allele. Although the population of DRB3*016:01-carrying cattle showed significantly higher PVLs compared with cattle carrying other alleles, their individual PVLs were highly variable. Because of the simplicity and speed of this single-well assay, our method has the potential of being a suitable platform for the combined diagnosis of pathogen level and host biomarkers in other infectious diseases satisfying the two following characteristics of disease outcomes: (i) pathogen level acts as a critical maker of disease progression; and (ii) impactful disease-related host genetic biomarkers are already identified.

**IMPORTANCE** While pathogen-level quantification is an important diagnostic of disease severity and transmissibility, disease-related host biomarkers are also useful in predicting outcomes in infectious diseases. In this study, we demonstrate that combined proviral load (PVL) and host biomarker diagnostics can be used to detect bovine leukemia virus (BLV) infection, which has a negative economic impact on the cattle industry. We developed a fourplex droplet digital PCR assay for PVL of BLV and susceptible and resistant host genes named IPATS-BLV. IPATS-BLV has inherent merits in measuring PVL and identifying susceptible and resistant cattle with superior simplicity and speed because of a single-well assay. Our new laboratory technique contributes to strengthening risk-based herd management used to control within-herd BLV transmission. Furthermore, this assay design potentially improves the diagnostics of other infectious diseases by combining the pathogen level and disease-related host genetic biomarker to predict disease outcomes.

## INTRODUCTION

Overcoming the threat of infectious disease requires an accurate risk assessment of the disease severity for individuals. In viral infections, the viral load is diagnostically important because it acts as an indicator of disease severity (1–3) and transmissibility (4–6). In addition to the usefulness of viral load, an identification of disease-related host biomarkers is also important because it leads to a prediction of disease outcomes. Human leukocyte antigen (HLA) (major histocompatibility complex [MHC] in humans) proteins on the surface of cells are involved with the regulation of innate immunity and antigen presentation (7, 8). The HLA haplotype is informative for predicting the strength of an individual’s immune responses against pathogens and is a useful indicator of disease susceptibility (9, 10). The impact of differing immune capacities against viral replication could result in the emergence of a rare population with a high transmissibility to others (superspreaders). Additionally, high-immune capacity can suppress an individual’s viral load to levels undetectable by diagnostic testing (e.g., elite controllers in human immunodeficiency virus studies). Determining both the viral load and HLA haplotype has the benefit of accurately identifying infection susceptible/severe disease patients and infection-resistant/mild disease patients. Such information supports the prioritization of intensive medicine and vaccination for the at-risk population.

Improved diagnostics have significantly contributed to tackling the problems of livestock infectious diseases. The eradication of highly contagious diseases, such as foot and mouth diseases (11) and African swine fever (12), and of chronic, untreatable diseases, such as paratuberculosis (Johne’s disease) (13) and bovine leukemia virus (BLV) infection (14), is an unavoidable challenge to ensuring future food production. These diseases are listed as notifiable terrestrial animal diseases by the World Organization for Animal Health (15). The latter diseases are difficult to control because of their silent spread, owing to the lack of clinical signs, and the unfeasibility of culling all infected animals, owing to the high prevalence worldwide (16, 17). To control these diseases while preserving as many animals as possible, identifying and isolating superspreader animals and maintaining disease-resistant animals via selective breeding are reasonable approaches.

BLV belongs to the genus *Deltaretrovirus* in the *Retroviridae* family, and it has a genomic structure and properties similar to those of human T-lymphotropic virus type 1 (18). BLV causes production issues in livestock farms by reducing the milk and meat productivity of infected cattle (19, 20). Furthermore, just under 10% of BLV-infected cattle develop a malignant B-cell lymphoma called enzootic bovine leukosis (EBL), which is a lifelong infection (21). As there are no effective treatments or vaccines for BLV infection (22), an appropriate intervention to prevent the spread of this virus is needed. BLV transmits via the direct transfer of infected blood, so the proviral load (PVL) is a determinant of transmissibility.

Previous research revealed an association between *exon 2* of the bovine MHC (*BoLA*)-*DRB3* gene (*DRB3*) and the BLV PVL. In the Japanese Black species of cattle, having DRB3*016:01 is associated with a high PVL (HPL) of BLV; thus, this allele is considered to be a BLV susceptibility gene (23, 24). In contrast, having DRB3*009:02 is strongly associated with a low PVL (LPL) of BLV in the Japanese Black and Holstein species of cattle; thus, this allele is considered to be a BLV resistance gene (23–29). To provide a method for identifying BLV superspreaders by PVL quantification and BLV-susceptible/resistant gene-possessing cattle by allele typing more easily and rapidly, we developed a single-well droplet digital PCR (ddPCR)-based measurement system for the BLV PVL, DRB3*016:01 allele, and DRB3*009:02 allele.

## RESULTS

### Single-well measurement of BLV PVL, DRB3*016:01, and DRB3*009:02.

**FIG 1:**
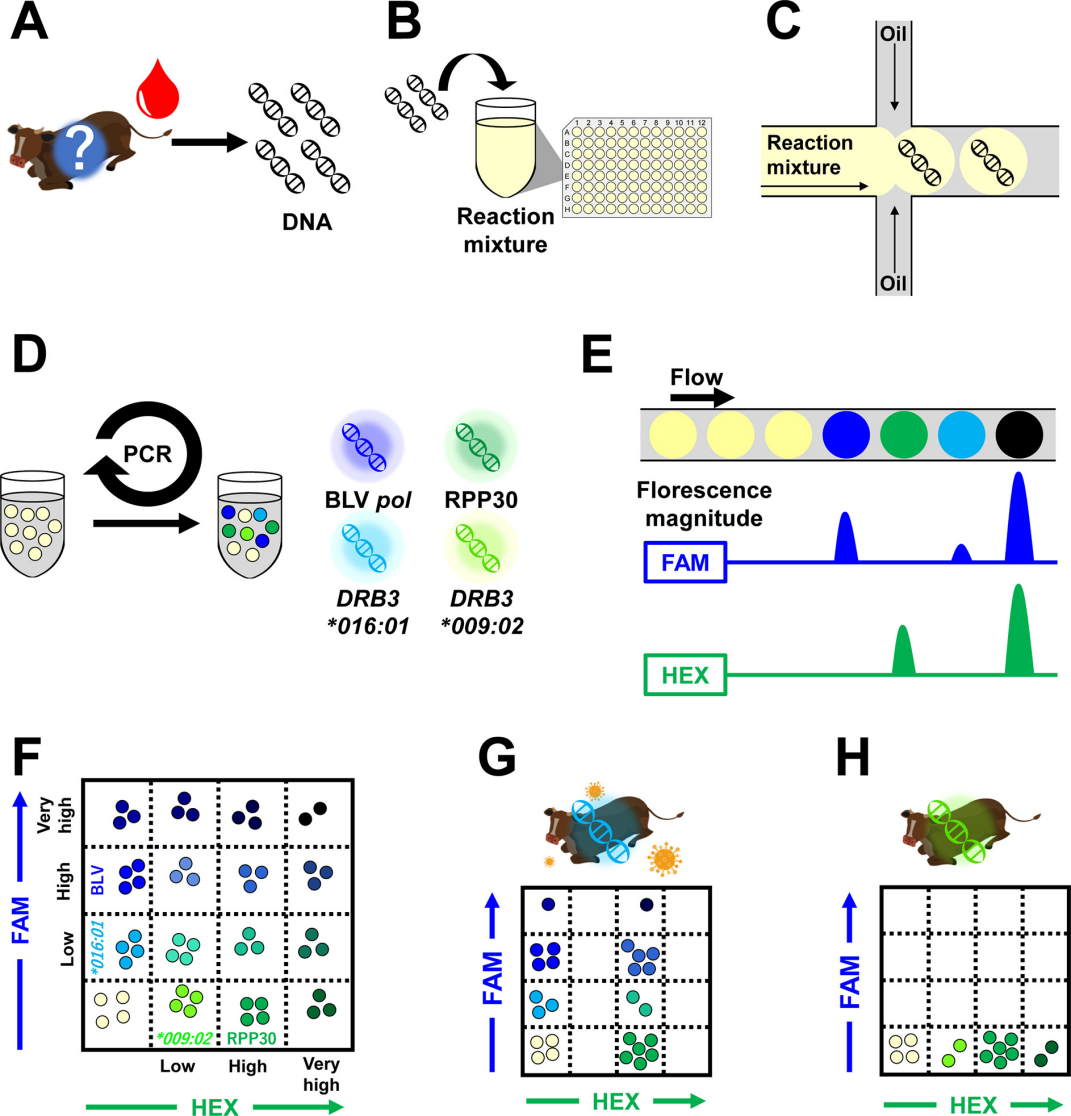
Workflow of IPATS-BLV. The work flow is indicated from A to F. (A) DNA extraction from bovine whole blood. (B) Addition of DNA samples to the reaction mixture. (C) Generation of droplets for partitioning the sample DNA. (D) Fourplex TaqMan Assay of the droplets. (E) Determination of the florescence magnitude. (F) 2D amplitude indicating the position of droplet clusters according to the fluorescence magnitude. (G) 2D amplitude pattern of DRB3*016:01-carrying cattle with a HPL of BLV. (H) 2D amplitude pattern of DRB3*009:02-carrying cattle with an undetectable PVL of BLV.

This study aimed to design a method for easily and rapidly quantifying BLV PVL and identifying BLV-susceptible DRB3*016:01-carrying cattle and BLV-resistant DRB3*009:02-carrying cattle. We developed a fourplex ddPCR targeting the BLV *pol* gene, DRB3*016:01 allele, DRB3*009:02 allele, and housekeeping RPP30 gene, named IPATS (Identifying Pathogen and Allele Type Simultaneously)-BLV (Fig. 1A to H). This assay consists of a multiplex TaqMan assay using seven primers, including two locked nucleic acid (LNA) primers and four TaqMan probes in a single well (Table S1 and S2 in the supplemental material). By modulating the amplicon length and primer/probe concentration in the reaction mixture, we succeeded in detecting two targets in the same color with separate fluorescence magnitudes in the PCR-positive droplet (30, 31). When a droplet contains a DRB3*016:01 allele or/and DRB3*009:02 allele, which we set to be detected by a low-concentration probe (approximately 200 bp of amplicon), the droplet exhibits a low level of FAM or/and HEX color, respectively, in our TaqMan assay. When a droplet contains a BLV *pol* gene or/and RPP30, which we set to be detected by a high-concentration probe (approximately 100 bp of amplicon), the droplet exhibits a high level of FAM or/and HEX color, respectively, in our TaqMan assay. When a droplet contains both DRB3*016:01 and the BLV *pol* gene (i.e., low and high levels of FAM color) or DRB3*009:02 and RPP30 (i.e., low and high levels of HEX color), a cluster showing a very high level of color is observed (Fig. 1D to F). This assay visualizes the properties of BLV PVL, DRB3*016:01 allele presence, and DRB3*009:02 allele presence in samples via the FAM and HEX amplitude cluster patterns of droplets (Fig. 1F). We used the percentage of BLV-infected cells as an indicator of the BLV PVL. We could calculate the percentage of BLV-infected cells by dividing the number of BLV-positive droplets by half of the number of RPP30-positive droplets. Furthermore, this assay can determine the homozygosity or heterozygosity of DRB3*016:01 and DRB3*009:02 by dividing the number of DRB3*016:01/DRB3*009:02-positive droplets by the number of RPP30-positive droplets.

10.1128/msphere.00493-22.1 TABLE S1 IPATS-BLV primers and probes. Download Table S1, DOCX file, 0.01 MB. Copyright © 2023 Notsu et al. 2023 Notsu et al. https://creativecommons.org/licenses/by/4.0/ This content is distributed under the terms of the Creative Commons Attribution 4.0 International license.

10.1128/msphere.00493-22.2 TABLE S2 Composition of IPATS-BLV reaction mixture. Download Table S2, DOCX file, 0.01 MB. Copyright © 2023 Notsu et al. 2023 Notsu et al. https://creativecommons.org/licenses/by/4.0/ This content is distributed under the terms of the Creative Commons Attribution 4.0 International license.

**FIG 2:**
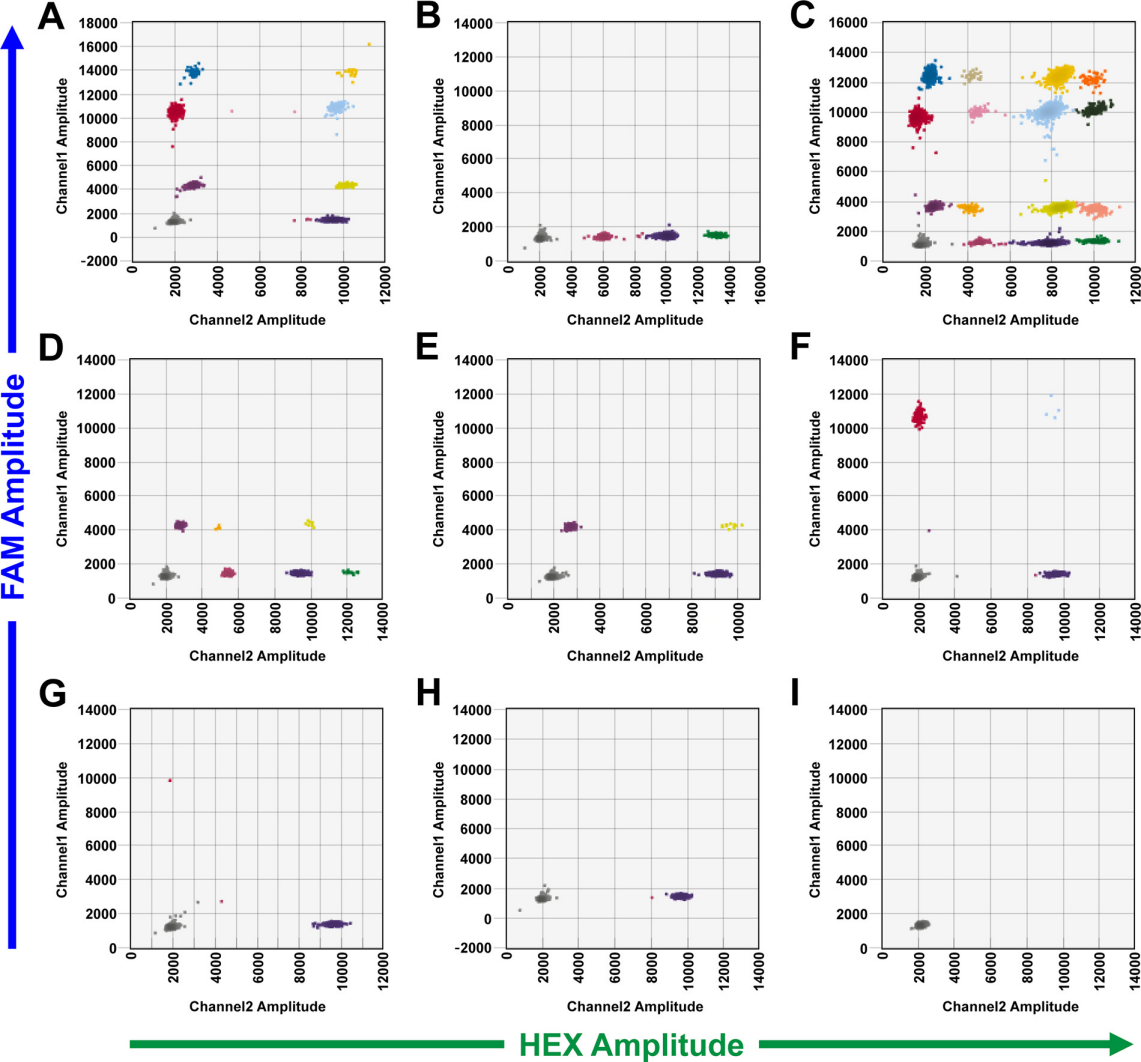
Cluster patterns in IPATS-BLV 2D amplitudes. Cluster patterns of IPATS-BLV of eight cattle with different possession of DRB3*016:01, DRB3*009:02, and BLV PVL and water are shown. Each droplet produces each different FAM and HEX fluorescence magnitude in TaqMan assay, reflecting a presence of targeting genes within droplet. Droplets makes clusters according to the similarity of fluorescence magnitude. The divisions of clusters are indicated by different color of droplets. (A) DRB3*016:01-carrying cattle with a HPL of BLV. (B) DRB3*009:02-carrying cattle with an undetectable BLV PVL. (C) Mixed population of DRB3*016:01/*015:01-carrying cattle with a HPL of BLV and DRB3*009:02/*015:01-carrying cattle (presumably DRB3*016:01/*009:02 heterozygous cattle with a detectable BLV PVL). (D) DRB3*016:01/*009:02-carrying cattle with an undetectable BLV PVL. (E) DRB3*016:01-carrying cattle with an undetectable BLV PVL. (F) Other allele-carrying cattle with a HPL of BLV. (G) Other allele-carrying cattle with a LPL of BLV. (H) Other allele-carrying cattle with an undetectable BLV PVL. (I) Water.

As shown in Fig. 2A to I, IPATS-BLV produces a variety of cluster patterns of FAM and HEX fluorescence intensity in two-dimensional (2D) amplitude. DRB3*016:01-carrying cattle with a HPL of BLV produce the cluster patterns shown in Fig. 2A and C (Fig. 2C displays the pattern produced by DRB3*016:01/*009:02-carrying cattle with a HPL of BLV). In contrast, DRB3*009:02-carrying cattle with an undetectable BLV PVL produce the cluster patterns shown in Fig. 2B and D (Fig. 2D displays the pattern produced by DRB3*016:01/*009:02-carrying cattle with an undetectable BLV PVL). DRB3*016:01-carrying cattle with an undetectable PVL of BLV produce the pattern shown in Fig. 2E. When cattle carry neither the DRB3*016:01 allele nor the DRB3*009:02 allele, those with a HPL, a LPL, or an undetectable BLV PVL produce the cluster patterns shown in Fig. 2F, G, and H, respectively. Figure 2I displays the pattern produced by water that is negative for all the target genes. A one-dimensional amplitude of these patterns is provided in Fig. S1A to I.

10.1128/msphere.00493-22.6 FIG S1 Cluster patterns in 1D amplitude of IPATS-BLV, related to Fig. 2. Above and below indicates FAM and HEX amplitude, respectively. (A) DRB3*016:01-carrying cattle with a HPL of BLV. (B) DRB3*009:02-carrying cattle with an undetectable BLV PVL. (C) Mixed population of DRB3*016:01/*015:01-carrying cattle with a HPL of BLV and DRB3*009:02/*015:01-carrying cattle (presumably DRB3*016:01/*009:02 heterozygous cattle with a detectable BLV PVL). (D) DRB3*016:01/*009:02-carrying cattle with an undetectable BLV PVL. (E) DRB3*016:01-carrying cattle with an undetectable BLV PVL. (F) Other allele-carrying cattle with a HPL of BLV. (G) Other allele-carrying cattle with a LPL of BLV. (H) Other allele-carrying cattle with an undetectable BLV PVL. (I) Water. Download FIG S1, TIF file, 0.2 MB. Copyright © 2023 Notsu et al. 2023 Notsu et al. https://creativecommons.org/licenses/by/4.0/ This content is distributed under the terms of the Creative Commons Attribution 4.0 International license.

### Digital allele typing with high accuracy.

To assess the accuracy of the DRB3*016:01 and DRB3*009:02 genotyping by our new method, we performed IPATS-BLV on 58 bovine genomic DNA samples with *DRB3* allele variations. These samples were previously genotyped using combined PCR-restriction fragment length polymorphism (RFLP) sequencing (26, 32, 33). A total of 21 *DRB3* alleles were identified by this previous analysis (Table S3). Among these samples, IPATS-BLV successfully discriminated seven samples with DRB3*016:01 alleles and 14 samples with DRB3*009:02 alleles by calculating the ratio of the number of DRB3*016:01-positive (DRB3*016:01 ratio) and DRB3*009:02-positive (DRB3*009:02 ratio) droplets to the number of RPP30-positive droplets (Table 1). Five of these samples were DRB3*016:01/*009:02 heterozygous. The DRB3*016:01 ratio of these seven samples with DRB3*016:01 was 0.4646 (standard error [SE]: ±0.01087). The DRB3*009:02 ratio of these 14 samples with DRB3*009:02 was 0.4658 (SE: ±0.00779). Because we rounded the values of the DRB3*016:01 and DRB3*009:02 ratios of samples carrying other alleles to two decimal places to suppress the effect of noise, all these samples had values of 0.0 for their ratios, except for a sample carrying heterozygous DRB3*037:01/*044:01 (Table S3, yellow highlight), which had a DRB3*016:01 ratio value of 0.2. Thus, IPATS-BLV showed the complete agreement of DRB3*016:01 and DRB3*009:02 genotyping with combined PCR-RFLP sequencing in our field samples. For the *DRB3* alleles that have similar sequences with DRB3*016:01 and DRB3*009:02, we tested using plasmid DNA because such variants are seldom found from the field. Our results for DRB3*071:01, DRB3*100:01, and DRB3*100:05 plasmid DNAs, which have similar sequences with DRB3*016:01 (Table S4), showed inadequate resolution of fluorescence magnitude in FAM low clusters (Fig. S2B to D, respectively). We also tested DRB3*009:01 and DRB3*024:01 plasmid DNAs because they have similar sequences to DRB3*009:02 (Table S4). DRB3*009:01 plasmid did not show any signal (Fig. S2F). DRB3*024:01 showed inadequate resolution of fluorescence magnitude in HEX low clusters (Fig. S2G). Thus, we concluded IPATS-BLV is capable of discriminating these alleles by judging PCR noise or negative patterns using cluster patterns of 2D amplitude.

**TABLE 1:** Comparison of the allele detectability of IPATS-BLV and combined PCR-RFLP sequencing

	Combined PCR-RFLP sequencing			
IPATS-BLV	DRB3*016:01	DRB3*009:02	DRB3*016:01/*009:02	Other alleles
DRB3*016:01/other allele^*a*^	2	0	0	0
DRB3*009:02/other allele^*b*^	0	9	0	0
DRB3*016:01/*009:02	0	0	5	0
Other alleles	0	0	0	42

a Except DRB3*009:02.

b Except DRB3*016:01.

10.1128/msphere.00493-22.3 TABLE S3 Genotyping of various alleles using IPATS-BLV, related to Table 1. Download Table S3, DOCX file, 0.02 MB. Copyright © 2023 Notsu et al. 2023 Notsu et al. https://creativecommons.org/licenses/by/4.0/ This content is distributed under the terms of the Creative Commons Attribution 4.0 International license.

10.1128/msphere.00493-22.4 TABLE S4 Sequences of tested alleles that have similar sequences with DRB3*016:01 and DRB3*009:02 at IPATS-BLV primer/probe sites. Download Table S4, DOCX file, 0.02 MB. Copyright © 2023 Notsu et al. 2023 Notsu et al. https://creativecommons.org/licenses/by/4.0/ This content is distributed under the terms of the Creative Commons Attribution 4.0 International license.

10.1128/msphere.00493-22.7 FIG S2 Discrimination of *DRB3* alleles that has similar sequences with DRB3*016:01 and DRB3*009:02. (A) Control pattern of DRB3*016:01 with codetection of DRB3*009:02/*015:01-carrying cattle with an undetectable BLV PVL, see Fig. 2D. The location of clusters of DRB3*016:01-positive droplets is indicated by red outline. (B) DRB3*071:01 plasmid with codetection of DRB3*009:02/*015:01-carrying cattle with an undetectable BLV PVL. (C) DRB3*100:01 plasmid with codetection of DRB3*009:02/*015:01-carrying cattle with an undetectable BLV PVL. (D) DRB3*100:05 plasmid with codetection of DRB3*009:02/*015:01-carrying cattle with an undetectable BLV PVL. (E) Control pattern of DRB3*009:02 with codetection of DRB3*016:01/*015:01-carrying cattle with a HPL of BLV, see Fig. 2C. The location of clusters of DRB3*009:02-positive droplets is indicated by red outline. (F) DRB3*009:01 plasmid with codetection of DRB3*009:02/*015:01-carrying cattle with a HPL of BLV. (G) DRB3*024:01 plasmid with codetection of DRB3*009:02/*015:01-carrying cattle with a HPL of BLV. Download FIG S2, TIF file, 0.3 MB. Copyright © 2023 Notsu et al. 2023 Notsu et al. https://creativecommons.org/licenses/by/4.0/ This content is distributed under the terms of the Creative Commons Attribution 4.0 International license.

### BLV infection diagnostic performance of IPATS-BLV is comparable with that of other diagnostic methods.

We first evaluated the BLV infection diagnostic performance of IPATS-BLV by comparing it with that of the anti-gp51 antibody ELISA. We performed both the ELISA and IPATS-BLV for 65 samples with an unknown infectious status. We qualitatively compared the ELISA-positive/negative results versus the IPATS-BLV-positive/negative results. As shown in Table 2, 27 samples were identified as BLV positive and 33 samples as BLV negative by both assays. One sample was identified as BLV positive by IPATS-BLV but as BLV negative by ELISA; this discrepancy could result from a sample taken during the initial phase of BLV infection. Four samples were identified as BLV-negative by IPATS-BLV but as BLV positive by ELISA. This result might indicate that these cattle were capable of suppressing an increase in the BLV PVL. Among these cattle, one was identified as carrying the DRB3*009:02 allele. The kappa value between the IPATS-BLV and ELISA was 0.8452 (SE ±0.1235).

**TABLE 2:** Comparison of the BLV detectability of IPATS-BLV and ELISA

	Anti-gp51-ELISA	
IPATS-BLV	Positive	Negative
Positive	27	1
Negative	4	33

**FIG 3:**
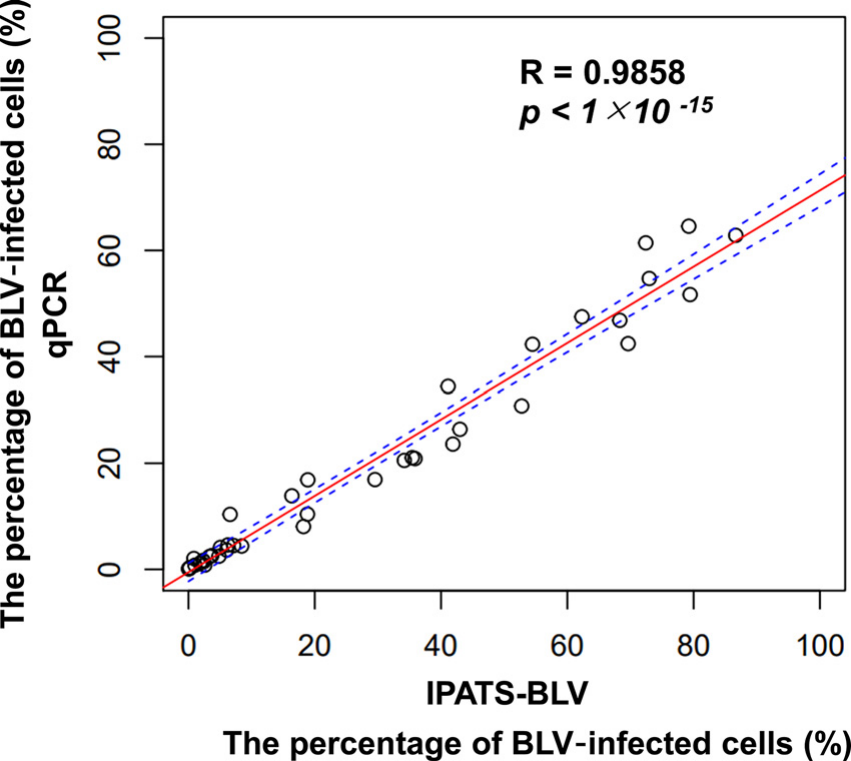
Correlation analysis of the measurement of the percentage of BLV-infected cells between IPATS-BLV and qPCR. The red line and blue dotted line indicate the linear model and 95% confidence interval, respectively.

Next, we evaluated the accuracy of the measurement of the percentage of BLV-infected cells by IPATS-BLV via a comparison with quanatitative PCR (qPCR). We found a strong correlation (Pearson’s coefficient *R* = 0.9858, *P < *1 × 10^−15^) between these two assays, based on the measurement of 40 samples with variation in their percentage of BLV-infected cells (Fig. 3). Finally, we determined the limit of detection (LOD) of the percentage of BLV-infected cells in IPATS-BLV using DNA extracted from serially diluted whole blood of BLV-infected cattle. IPATS-BLV could detect BLV provirus from cattle in which 1.50 × 10^−1^ percent of cells were infected with BLV, which is comparable to the LOD of commercial qPCR for BLV provirus (Table 3).

**TABLE 3:** Comparison of the BLV LOD between qPCR and IPATS-BLV

	qPCR	IPATS-BLV	
Percentage of BLV-infected cells (%)	*C_T_*	No. of positive droplet	CNV2^*a*^
1.50			
Fraction 1	34.71	39	0.014444
Fraction 2	34.32	37	0.016897
Fraction 3	34.68	46	0.018678
1.50 × 10^−1^			
Fraction 1	38.08	5	0.001672
Fraction 2	38.3	3	0.001059
Fraction 3	39.52	6	0.002066
1.50 × 10^−2^			
Fraction 1	Undetected	1	0.000326
Fraction 2	40.65	0	NA^*b*^
Fraction 3	Undetected	0	NA^*b*^
1.50 × 10^−3^			
Fraction 1	Undetected	0	NA^*b*^
Fraction 2	Undetected	0	NA^*b*^
Fraction 3	Undetected	0	NA^*b*^

a BLV copy number per two RPP30 copies.

b Not available.

### Survey for the percentage of DRB3*016:01- and DRB3*009:02-carrying cattle and the impact of these alleles on the percentage of BLV-infected cells.

**FIG 4:**
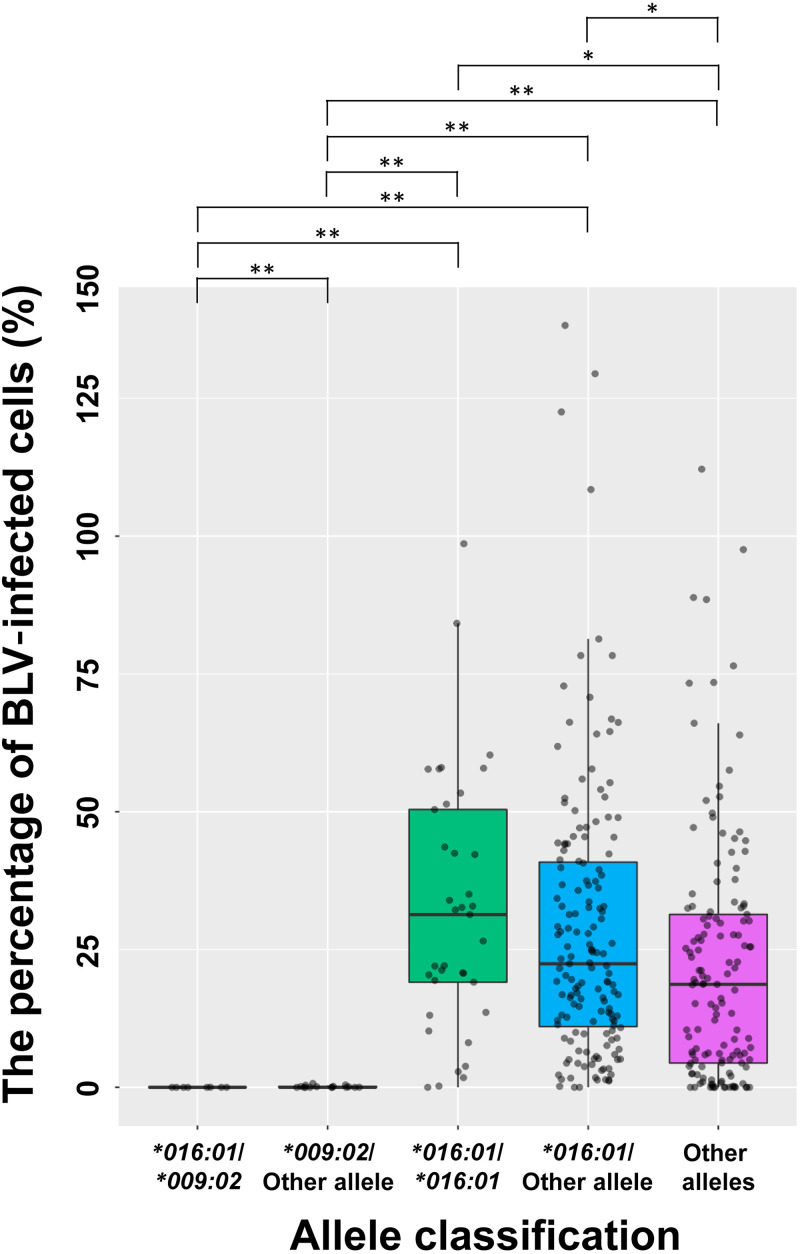
Comparison of the percentage of BLV-infected cells by allele classification. A box-and-whisker plot is shown. Box indicates 25th to 75th percentile of the range of the percentage of BLV-infected cells. Intermediate line in the box is the median. Dot represent each sample. *, *P < *0.05; **, *P < *0.0001.

A field survey of the percentage of DRB3*016:01- or DRB3*009:02-carrying cattle and the impact of these alleles on the BLV PVL was carried out in Miyazaki prefecture, Japan. First, we used an anti-gp51 ELISA to screen for BLV-infected cattle. Among 4,603 asymptomatic Japanese Black cattle from 1,394 farms, 353 cattle (7.7%) from 164 farms were identified as BLV positive by ELISA (“ELISA positive”). We then performed IPATS-BLV on samples from the 353 ELISA-positive cattle; 200 cattle (56.7%) and 24 cattle (6.8%) were found to carry DRB3*016:01 and DRB3*009:02, respectively. Before performing a comparison of the percentage of BLV-infected cells, we classified these cattle into the following five groups: DRB3*016:01/*009:02 heterozygous (*n* = 8), DRB3*009:02/other allele heterozygous (*n* = 16), DRB3*016:01/*016:01 (DRB3*016:01 homozygous) (*n* = 37), DRB3*016:01/other allele heterozygous (*n* = 155), and other alleles (*n* = 137) (Fig. 4). The 37 DRB3*016:01 homozygous cattle showed an average DRB3*016:01 ratio of 0.9930 (SE ±0.014965). Cattle with a DRB3*009:02 allele had a significantly lower percentage of BLV-infected cells compared with the other groups, even when the cattle were heterozygous for the BLV-susceptible DRB3*016:01 allele. Although cattle with a DRB3*016:01 allele had a statistically significantly higher percentage of BLV-infected cells compared with other allele-carrying cattle, their PVLs varied widely.

## DISCUSSION

The IPATS-BLV method provides an absolute DNA quantification of the BLV *pol* gene, BLV-susceptible DRB3*016:01 allele, BLV-resistant DRB3*009:02 allele, and RPP30 by using a fourplex ddPCR. IPATS-BLV was demonstrated to accurately measure the percentage of BLV-infected cells and provide highly sensitive and specific allele typing that discriminates between homozygous and heterozygous carriers, all in a single-well reaction. We found that cattle carrying the BLV-resistant DRB3*009:02 allele had a strong ability to maintain the PVL of BLV at a low or undetectable level. In contrast, DRB3*016:01-carrying cattle were found to have a relatively higher percentage of BLV-infected cells compared with other allele-carrying cattle.

Here, we demonstrated the allelic impact of the previously identified BLV-resistant DRB3*009:02 allele and BLV-susceptible DRB3*016:01 allele on the BLV PVL, as shown in Fig. 4. DRB3*009:02-carrying cattle had a low/undetectable level of BLV PVL, even when their other allele was the BLV-susceptible DRB3*016:01 allele. This result is supported by previous studies, indicating a strong association between DRB3*009:02 and a low/undetectable PVL of BLV under the consideration of allele heterozygosity (23, 26). However, not all DRB3*009:02-carrying cattle are BLV resistant (34). It seems that BLV resistance is determined by not only the *DRB3* allelic effect but also other factors, such as species and climate. One advantage of IPATS-BLV is that it identifies BLV elite controllers based on both DRB3*009:02 and an undetectable BLV PVL.

DRB3*016:01-carrying cattle had a significantly higher PVL of BLV compared with cattle with other alleles. This is supported by a previous study indicating that the percentage of BLV HPL cattle was higher among the group of DRB3*016:01-carrying cattle (24). However, our results also suggest that the PVL of DRB3*016:01-carrying cattle varies widely. BLV-susceptible DRB3*016:01 does not strongly associate with BLV HPL, unlike the strong association between DRB3*009:02 and low/undetectable BLV PVL regardless of allele heterozygosity. Importantly, BLV-infected cattle with DRB3*016:01 homozygous, not heterozygous, were significantly associated with disease progression to lymphoma (23). Identifying cattle with DRB3*016:01 homozygous is important in breeding because they certainly transmit that allele to their offspring. Thus, discrimination of DRB3*016:01 homozygous from heterozygous carriers, which is one of the inherent abilities of IPATS-BLV, contributes to an improvement of selective breeding by avoiding the use of DRB3*016:01 homozygous cattle. Currently, there is limited knowledge of the susceptibility of DRB3*016:01 to other diseases. Further investigation into associating this allele with other diseases could provide the basis for the justification of removing cattle with this allele.

The simultaneous detection of pathogens and host biomarkers contributes to strengthening the control of livestock infectious diseases. Because there are presently no vaccines or effective treatments for BLV infection, prevention is the only available countermeasure. BLV was previously eliminated in some countries in Europe via the identification and stamping out of infected animals and the restriction of between-farm cattle movement from infected farms (35, 36). As the BLV PVL varies by individual, depending on the virus-host interaction and other factors, not all infected cattle pose a risk of transmitting BLV to other cattle. Recently, BLV control based on the PVL has been implemented under the presumption that cattle with a LPL have low or no risk of BLV transmission (37–39). In addition to viral factors, host factors such as the *DRB3* haplotype have also received focus as an indicator of BLV disease susceptibility (40). Several studies identified some *DRB3* alleles as being associated with a LPL, including the strongly resistant DRB3*009:02 (23–29). The identification of BLV elite controllers will be useful in disrupting the chain of BLV transmission (37). Despite the benefit of herd management conducted based on both PVL and *DRB3* haplotype, it is too time consuming to implement if PVL measurement and allele typing need to be performed independently. Our newly developed method allows these data to be obtained more easily and rapidly and could be further applied to a high-throughput diagnosis. The power of IPATS-BLV opens a new avenue of BLV control by permitting the consideration of both PVL and genetic susceptibility.

Disease control using resistant animals has an aspect of providing assurance for food safety. Because of the genetic variation in susceptibility to infectious diseases among species, derived from coevolution with pathogens (41, 42), a population of livestock possessing the power of disease resistance should exist latently everywhere. As selective breeding is an applied use of natural resources, there is no need to evaluate its adverse health effects on humans, unlike products of genome engineering. In the case of genetically modified crops, commercialization requires 13 years from project development and 35.01 million U.S. dollars for the cost of regulatory safety assessment and securing global registration and authorizations. Notably, it takes 5 to 7 years to perform the safety evaluations and obtain regulatory approval (43). Ethical problems are also unavoidable when applying genome engineering to animals. Taken together, despite the advantage of the customizability of genome engineering for livestock, there is a bottleneck in implementing this approach. Genetic selection, which is already performed largely in marine (44), forest (45), and livestock agriculture (46), is a feasible alternative to genome engineering. This technique is ready to use when the equipment for selective breeding and diagnostics is available.

Consideration of both pathogen levels and host biomarkers has the potential for improving decision-making regarding the treatment and prevention of infectious diseases by providing a deeper understanding of individual infection. For example, septic shock outcomes can be successfully predicted by merging information about the quantity of bacteria and cytokines in a patient (47). Regarding the current outbreak of severe acute respiratory syndrome coronavirus 2 (SARS-CoV-2) infection, researchers are discussing that HLA typing with viral diagnosis could improve the assessment of disease severity and allow high-risk individuals to be prioritized for vaccination (48). Such concepts contribute to improving preventive veterinary medicine by supporting appropriate herd management. Even when there are effective treatments and vaccinations for some threatening infectious diseases, some countries have a distribution bottleneck for these pharmacologic compounds owing to complex matters, including supply chain and equipment (49). Managing animals according to their current and future risk of disease transmissibility results in the best usage of available bioresources to suppress the damage from infectious diseases. Therefore, we expect the power of improved diagnostics to contribute to sustainable production from livestock in the future.

Some limitations of this study must be discussed. First, DRB3*009:02-carrying cattle with an undetectable BLV PVL could be either a BLV elite controller or an uninfected animal. We recommend the use of IPATS-BLV in combination with an antibody detection method, such as an ELISA. Second, DRB3*009:02-carrying cattle can have detectable BLV PVL in the initial phase of BLV infection (50). Thus, the determination of the BLV elite controller should be conducted by testing the PVL several times. Finally, we did not test all *DRB3* variants in the experiments for the discriminability of DRB3*016:01 and DRB3*009:02 in IPATS-BLV because of the unavailability of obtaining samples of these alleles-carrying cattle. We note that tested the 26 *DRB3* alleles (including plasmids) in this study occupy the majority of the population in Japanese Black and Holstein species; 90.7% of Japanese Black and 96.7% of Holstein species in Japan (51), 98.2% of Holstein species in Korea (52), 72.6% of beef cattle in the United States (53), and 92.7% of Holstein species in South America region (54). In the case that the sample population has an uninvestigated background of *DRB3* possession, we recommend confirming DRB3*016:01 and DRB3*009:02 by both IPATS-BLV and sequencing to confirm the presence of minor *DRB3* alleles. We believe IPATS-BLV is useful for the screening of cattle possibly possessing these alleles because this assay does not have a risk of false negatives.

In conclusion, IPATS is an easy and rapid platform with which to measure pathogen levels and disease-related host biomarkers and one that is already identified. It potentially provides strengthened diagnostics that consider both the actual disease severity/transmissibility and disease susceptibility of the host. Such an approach has the potential to become a key tool for next-generation human and veterinary medicine.

## MATERIALS AND METHODS

### IPATS-BLV assay design.

We designed a fourplex ddPCR based on BLV proviral DNA, DRB3*009:02, DRB3*016:01, and RPP30-TaqMan Assay (Fig. 1A to F). To address the limited number of channels in our commercial ddPCR system (e.g., QX200 Droplet Digital PCR system; Bio-Rad, Hercules, CA, USA), we modulated the amplicon length and primer/probe concentration in the reaction mixture to enable the separation of different targets within the same color (30, 31). We set the FAM low, FAM high, HEX low, and HEX high channels to DRB3*016:01, BLV *pol* gene, DRB3*009:02, and RPP30, respectively.

### Primer/probe.

We obtained 382 sequences of *DRB3 exon 2* (*DRB3.2*) alleles from the IPD-MHC database v. 3.6.0.1 (downloaded on 16 June 2021; https://www.ebi.ac.uk/ipd/mhc/). For DRB3*009:02, we designed allele-specific primers and probe via minor modification of a previously developed DRB3*009:02-TaqMan assay (32). To discriminate DRB3*016:01, we designed a DRB3*016:01-specific forward primer and probe. The DRB3*016:01-TaqMan assay shares the reverse primer for DRB3*009:02. One concern of this design was potential nonspecific reactions between the DRB3*016:01 primer/probe and DRB3*009:02 primer/probe. Thus, we recruited LNA primers to suppress the undesired amplification of untargeted alleles. To detect wild strains of BLV with sequence diversity, we designed primers and probes targeting a conserved region in the *pol* gene, as identified from a database of aligned sequences for 82 reported strains (Table S5). This database includes 72 strains of BLV genotype 1 (G1), which is currently dominant worldwide, one strain of G2, one strain of G4, three strains of G6, four strains of G9, and one strain of G10. The primers and probe target a position in the 3′-terminal end of the *pol* gene (Fig. S3) that is conserved except for an acceptable mismatch at the 5′ side of the forward primer in the par91 strain (accession no. LC080658). We added primers and probes for RPP30 into the reaction for housekeeping purposes. Table S1 indicates the sequences of the primers/probes. We purchased all these primers and probes, except for the LNA primers, from Eurofins Genomics (Tokyo, Japan). We purchased the LNA primers from Qiagen (Hilden, Germany).

10.1128/msphere.00493-22.5 TABLE S5 List of aligned BLV strains to determine conserved region. Download Table S5, DOCX file, 0.02 MB. Copyright © 2023 Notsu et al. 2023 Notsu et al. https://creativecommons.org/licenses/by/4.0/ This content is distributed under the terms of the Creative Commons Attribution 4.0 International license.

10.1128/msphere.00493-22.8 FIG S3 Mutation mapping in BLV pol gene and location of primers and probe. Nucleotides in at least one strain had mismatch are red colored in a sequence of reference strain (pvAN003, accension no. AP018024). Primers and probe position are indicated by blue and pink outlines, respectively. Download FIG S3, TIF file, 2.1 MB. Copyright © 2023 Notsu et al. 2023 Notsu et al. https://creativecommons.org/licenses/by/4.0/ This content is distributed under the terms of the Creative Commons Attribution 4.0 International license.

### IPATS-BLV.

We finalized the IPATS-BLV reaction in a 22-μL reaction mixture containing 14 μL of 2× ddPCR Supermix for Probes (Bio-Rad), 909 nM primers except for the RPP30 primers (DRB3*016:01-forward, DRB3*009:02 forward, DRB3*009:02 reverse, BLV *pol* 4527 forward, and BLV *pol* 4638 reverse), 455 nM RPP30 forward and reverse primers, 68 nM FAM-labeled DRB3*016:01 probe, 182 nM HEX-labeled DRB3*009:02 probe, 295 nM FAM-labeled BLV *pol* 4560 probe, and 364 nM HEX-labeled RPP30 probe, and the sample was adjusted to <35 ng and the necessary volume of water to reach 22 μL (Table S2). We emulsified the reaction mixture using an automated droplet generator (Bio-Rad) for partitioning into droplets in accordance with the manufacturer’s instructions. We performed PCR amplification according to the following amplification profile: 95°C for 10 min; 60 cycles of 94°C for 30 s and 58°C for 2 min; and 98°C for 10 min. The FAM and HEX fluorescence magnitude of each droplet was read using a QX200 Droplet Reader (Bio-Rad). The number of droplets in each cluster was quantified by automatically/manually setting the appropriate fluorescence amplitude thresholds using QX Manager Software Standard Edition, Version 1.2 (Bio-Rad). We calculated the percentage of BLV-infected cells using equation 1.

(1) $$\mathrm{The percentage of BLV-infected cells}=\frac{\mathrm{The number of BLV-positive droplets}}{\mathrm{The number of RPP}30\mathrm{- positive droplets}÷2\;}\;\times 100$$

By calculating the ratio of the number of allele-positive droplets to the number of housekeeping-positive droplets using equation 2, we successfully discriminated whether cattle carry homozygous or heterozygous target alleles.

(2) $$\mathrm{DRB}3^{*}\;016\text{:}01\;\left(\text{or }\mathrm{DRB}3^{*}\;009\text{:}02\right)\text{ ratio}=\frac{\text{The number of }\mathrm{DRB}3^{*}\;016\text{:}01\;\left(\text{or }\mathrm{DRB}3^{*}\;009\text{:}02\right)\text{-positive}\;\text{droplets}}{\text{The number of RPP}30\text{-positive}\;\text{droplets}}$$

Ratios of approximately 1 and 0.5 indicate homozygosity and heterozygosity of an allele, respectively.

### Accuracy of DRB3*016:01 and DRB3*009:02 genotyping.

To determine the accuracy of DRB3*016:01 and DRB3*009:02 genotyping in IPATS-BLV, we genotyped 58 bovine genomic DNAs with varied *DRB3* alleles by IPATS-BLV. These samples included 21 *DRB3* alleles (Table S3), according to the results of *DRB3* allele determination using combined PCR-RFLP sequencing methods (26, 32, 33). We determined the agreement of *DRB3.2* allele typing between combined PCR-RFLP sequencing and IPATS-BLV. Furthermore, to evaluate the discriminability of IPATS-BLV for sequence similar alleles to DRB3*016:01 and DRB3*009:02 that are rarely found in field samples, we used artificially synthesized DNA. The pEX-A2J2 vector plasmid DNAs containing the sequence of DRB3*071:01 (accession no. DQ834892), DRB3*0100:01 (accession no. LC455386), DRB3*100:05 (accession no. LC455481), and DRB3*024:01 (accession no. KF870403) were purchased from Eurofins Genomics. For DRB3*009:01, pcDNA3.1D/V5-His-TOPO vector (Invitrogen, Thermo Fisher Scientific, Waltham, MA, USA), which contains the DRB3*009:01 (accession no. MT890683) cDNA that was constructed previously, was used. The sequence of these alleles in IPATS-BLV primer/probe site is indicated in Table S4. In IPATS-BLV assay, 2 pg of DRB3*071:01, DRB3*0100:01, and DRB3*100:05 plasmids, which potentially react with the DRB3*016:01 primer/probe set, was tested in the reaction mixture containing 20 ng of genomic DNA of DRB3*009:02/*015:01-carrying cattle. In addition, 2 pg of DRB3*009:01 and DRB3*024:01 plasmids, which potentially react with the DRB3*009:02 primer/probe set, was tested in the reaction mixture containing 20 ng of genomic DNA of DRB3*016:01/*015:01-carrying cattle with a HPL of BLV.

### Agreement with commercial ELISA.

We judged the agreement of qualitative detectability of BLV-infected cattle of IPATS-BLV with a commercial anti-gp51 antibody ELISA kit (Nippon gene, Tokyo, Japan). In the experiment, we used 65 bovine blood samples of unknown BLV infectious status. We isolated plasma by centrifuging the samples for 10 min at 1000 × *g*. The ELISA was performed in accordance with the manufacturer’s instructions. We extracted genomic DNA from whole blood using a Wizard Genomic DNA purification kit (Promega, Madison, WI, USA) and then performed IPATS-BLV. We defined samples as ELISA positive if their value was higher than the cutoff S/*P* value and as IPATS-BLV-positive if more than one BLV-positive droplet was detected in the amplitude. We evaluated the consensus of ELISA positive/negative versus IPATS-BLV positive/negative by calculating a kappa value using software in epitools (accessed on 16 June 2022; http://epitools.ausvet.com.au).

### Quantification of the percentage of BLV-infected cells.

For the accuracy of the quantification of the percentage of infected cells in IPATS-BLV, we determined the correlation of measurement with a commercial qPCR kit (number RC202A; TaKaRa, Shiga, Japan). The commercial qPCR kit targeted the BLV *pol* gene and RPPH1 for housekeeping. We extracted genomic DNA samples from the whole blood of cattle using MagDEA Dx SV reagent (Precision System Science, Chiba, Japan) with an automated nucleic acid extraction system (magLEAD 12gC; Precision System Science) in accordance with the manufacturer’s instructions. Next, we performed qPCR in accordance with the manufacturer’s instructions using QuantStudio 3 system (Applied Biosystems, Thermo Fisher Scientific). We selected 40 samples satisfying the variation of the percentage of BLV-infected cells and performed IPATS-BLV on these samples. The strength of the correlation between qPCR and IPATS-BLV was determined using Pearson’s coefficient, calculated using R software v. 3.6.2 (www.r-project.org).

### LOD of BLV detection.

To determine the LOD of BLV detection in IPATS-BLV, we tested DNA samples extracted from a serial dilution series of whole blood from BLV-infected cattle. This animal carried 1.5% of BLV-infected cells (as confirmed using qPCR). We serially diluted the whole blood of this animal 10 times using whole blood from a BLV-uninfected animal. We confirmed the “uninfected” status of these cattle by both an undetectable PVL in a qPCR assay and the absence of anti-BLV gp51 antibody in an ELISA. We extracted genomic DNA from three fractions of each dilution using magLEAD 12gC. We performed both IPATS-BLV and qPCR to compare the LOD. In both assays, the sample DNA input in the reaction mixture was 20 ng.

### Field survey.

We performed a field survey for the percentage of DRB3*016:01- or DRB3*009:02-carrying cattle and the impact of these alleles on the BLV PVL. We targeted asymptomatic Japanese Black cattle in Miyazaki prefecture, Japan. Whole blood samples were collected from 4,603 cattle over 1,394 farms by veterinarians and sent to the University of Miyazaki. These samples were collected from May 2020 to July 2022. Anti-BLV gp51 antibody ELISAs were performed immediately to screen for BLV-infected cattle. We stored the whole blood of ELISA-positive samples at −20°C until their use in further analysis. We extracted the genomic DNA of ELISA-positive cattle using either the magLEAD 12gC or a MagMAX CORE nucleic acid purification kit (Thermo Fisher Scientific) with an automated nucleic acid extraction system (KingFisher Duo Prime; Thermo Fisher Scientific). We performed IPATS-BLV for DRB3*016:01, DRB3*009:02, and BLV PVL. We classified these samples into the following five groups: DRB3*016:01/*009:02, DRB3*009:02/other allele, DRB3*016:01 homozygous, DRB3*016:01/other allele, and other alleles groups before a comparison of the percentage of BLV-infected cells between groups. We used a pairwise Wilcoxon rank sum test with Bonferroni’s modification for determining the significance of differences between each group using R software. Differences with a *P* value of <0.05 were judged as statistically significant.
