# Correction for Notsu et al., “Identifying Pathogen and Allele Type Simultaneously in a Single Well Using Droplet Digital PCR”

**DOI:** 10.1128/msphere.00208-23

**Published:** 2023-06-13

**Authors:** Kosuke Notsu, El Daous Hala, Shuya Mitoma, Xinyue Wu, Junzo Norimine, Satoshi Sekiguchi

Volume 8, No. 1, e00493-22, 2023, http://doi.org/10.1128/msphere.00493-22. Page 7, Fig. 4: We found errors in the significance indicator lines of this figure. In particular, there is no significant difference of the percentage of BLV-infected cells between the *016:01/*009:02 group and *009:02/other allele group. Instead, there is a significant difference between the *016:01/*009:02 group and the other alleles group. We note that this does not affect the overall discussion and conclusions. The corrected Fig. 4 is shown below.

**Fig 4:**
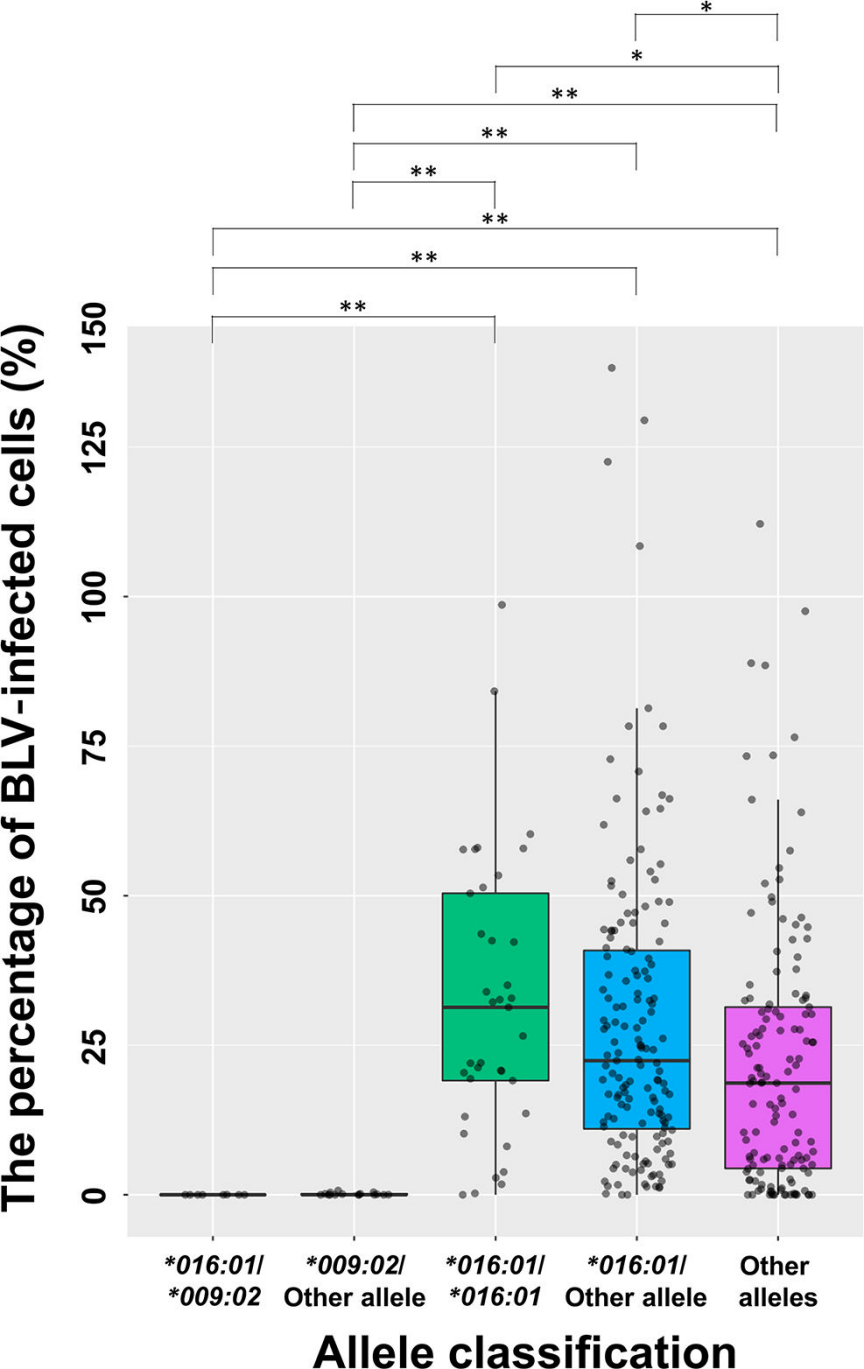
Comparison of the percentage of BLV-infected cells by allele classification. A box-and-whisker plot is shown. Box indicates 25th to 75th percentile of the range of the percentage of BLV-infected cells. Intermediate line in the box is the median. Dot represent each sample. *, *P < *0.05; **, *P < *0.0001.

