# Supplementary material for: Identifying Pathogen and Allele Type Simultaneously in a Single Well Using Droplet Digital PCR
Source: mSphere. 2023 Jan 10;8(1):e00493-22. doi: 10.1128/msphere.00493-22 (PMC9942588; doi:10.1128/msphere.00493-22)
Supplement: TABLE S1 [file msphere.00493-22-s0004.docx]

**Table S1.** IPATS-BLV primers and probes

| Primer/Probe | Target | Sequence 5' to 3' | Acc. No. of reference | Position |
| --- | --- | --- | --- | --- |
| *DRB3*016:01*-forward | *DRB3*016:01* | TTCGTGCGCTTCGA+T^a^ | AB610127.1 | 107-121 |
| *DRB3*016:01*-probe | *DRB3*016:01* | FAM-AGGACTTCCTGGAGGAGAA-MGB-Eclipse | AB610127.1 | 192-210 |
| *DRB3*009:02*-forward | *DRB3*009:02* | GTGCGGTTCCTGGA+G^a^ | AB610142.1 | 68-82 |
| *DRB3*009:02*-probe | *DRB3*009:02* | HEX-AGATCCTGGAGGAGAGGC-MGB-Eclipse | AB610142.1 | 195-212 |
| *DRB3*009:02*-reverse | *DRB3*016:01* and *DRB3*009:02* | CGCTGCACAGTGAAACTCTCA | AB610142.1 | 256-276 |
| BLV *pol* 4527-forward | BLV proviral DNA | GAACCCCACCTTCCCATGAC | AP018024.1 | 4,527-4,546 |
| BLV *pol* 4560-probe | BLV proviral DNA | FAM-CGAGCCCTCTGGACTCACAATC-BHQ1 | AP018024.1 | 4,560-4,581 |
| BLV *pol* 4638-reverse | BLV proviral DNA | GCCCTCTGAAATGACAGCAAG | AP018024.1 | 4,638-4658 |
| RPP30-forward | RPP30 | TGTTTCTGTTGGTCTGGTGTCC | NC_037353.1 | 12,515,902-12,515,923 |
| RPP30-probe | RPP30 | HEX-CGGCTGACTCTGGGCTGAA-MGB-Eclipse | NC_037353.1 | 12,515,925-12,515,943 |
| RPP30-reverse | RPP30 | CGGCCTTCGCATCACTTTC | NC_037353.1 | 12,515,984-12,516,002 |

^a^ +N indicates LNA.
