# Supplementary material for: Identifying Pathogen and Allele Type Simultaneously in a Single Well Using Droplet Digital PCR
Source: mSphere. 2023 Jan 10;8(1):e00493-22. doi: 10.1128/msphere.00493-22 (PMC9942588; doi:10.1128/msphere.00493-22)
Supplement: TABLE S2 [file msphere.00493-22-s0005.docx]

**Table S2.** Composition of IPATS-BLV reaction mixture

| Reagent/Primer/Probe/Sample | ×1 | Final concentration |
| --- | --- | --- |
| ddPCR™ Supermix for Probes (No dUTP) | 14 μl | - |
| *DRB3*016:01*-forward (40 μM) | 0.5 μl | 909 nM |
| *DRB3*016:01*-probe (10 μM) | 0.15 μl | 68 nM |
| *DRB3*009:02*-forward (40 μM) | 0.5 μl | 909 nM |
| *DRB3*009:02*-probe (10 μM*)* | 0.4 μl | 182 nM |
| *DRB3*009:02*-reverse (40 μM) | 0.5 μl | 909 nM |
| BLV *pol* 4527-forward (40 μM) | 0.5 μl | 909 nM |
| BLV *pol* 4560-probe (10 μM) | 0.65 μl | 295 nM |
| BLV *pol* 4638-reverse (40 μM) | 0.5 μl | 909 nM |
| RPP30-forward (20 μM) | 0.5 μl | 455 nM |
| RPP30-probe (10 μM) | 0.8 μl | 364 nM |
| RPP30-reverse (20 μM) | 0.5 μl | 455 nM |
| Water | 0.5 μl | - |
| DNA Sample (< 35 ng in reaction mixture) | 2 μl | - |
| Total | 22 μl | - |
