# Supplementary figures and images for: Identifying Pathogen and Allele Type Simultaneously in a Single Well Using Droplet Digital PCR
Source: mSphere. 2023 Jan 10;8(1):e00493-22. doi: 10.1128/msphere.00493-22 (PMC9942588; doi:10.1128/msphere.00493-22)

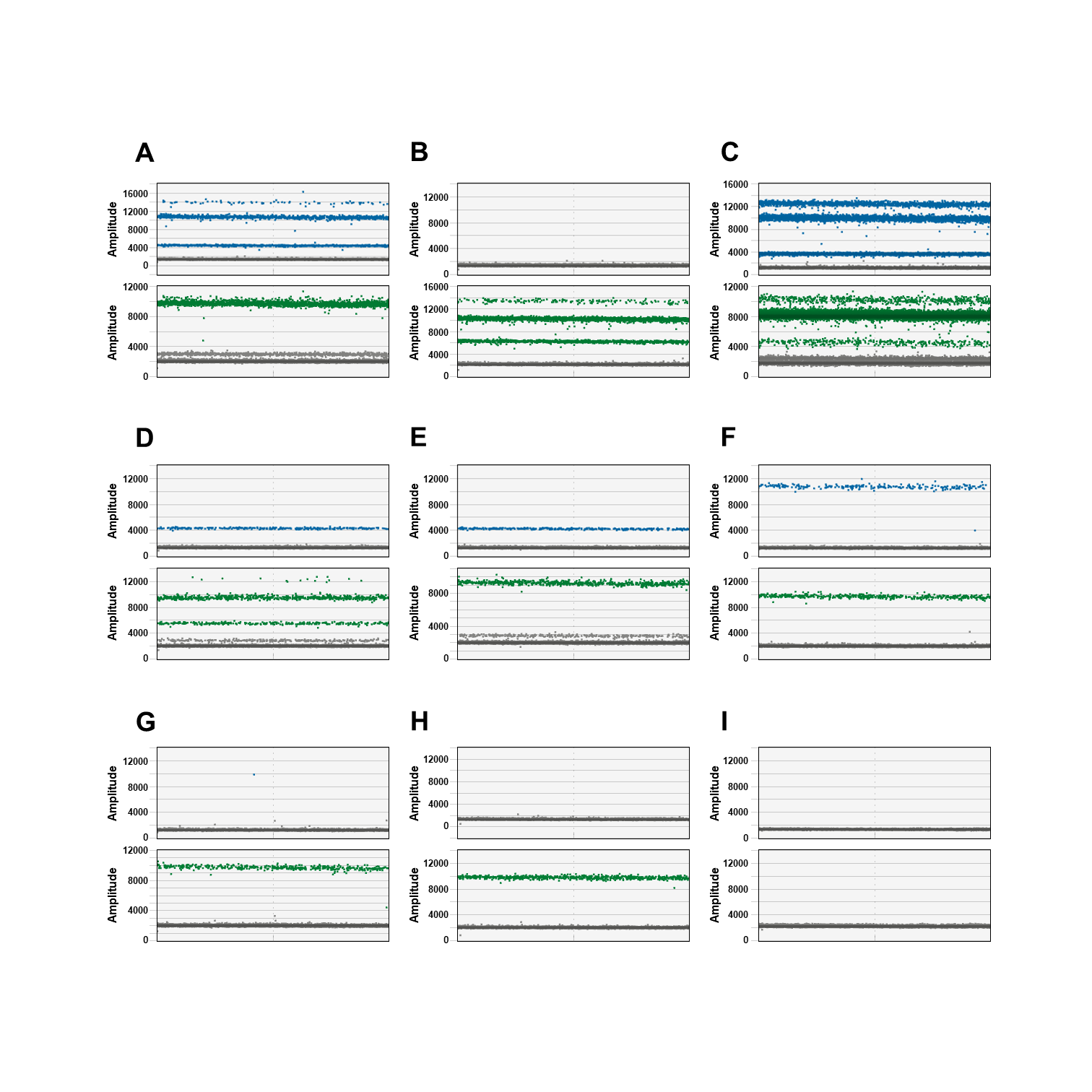

Supplement: FIG S1 [file msphere.00493-22-s0001.tif]

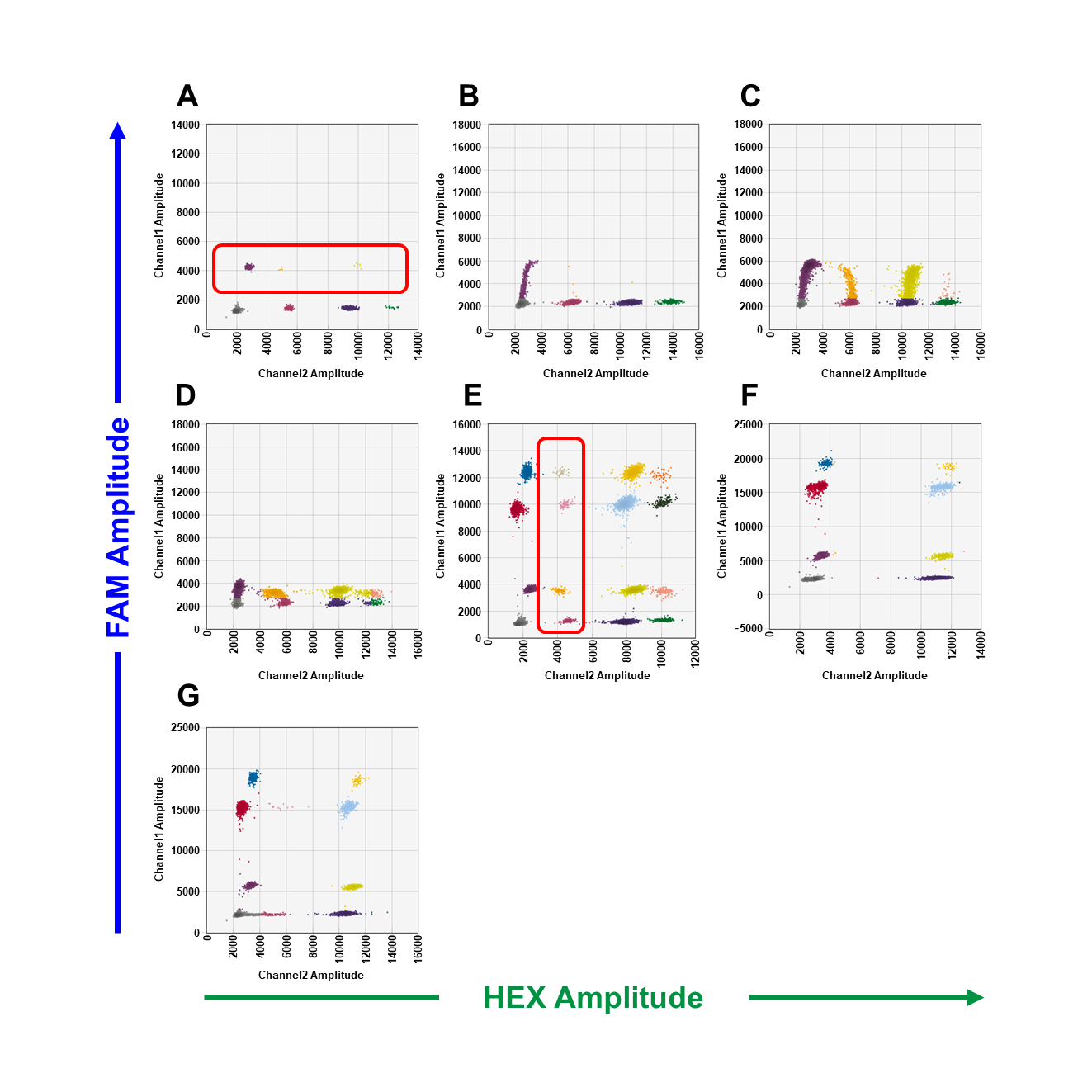

Supplement: FIG S2 [file msphere.00493-22-s0002.tif]

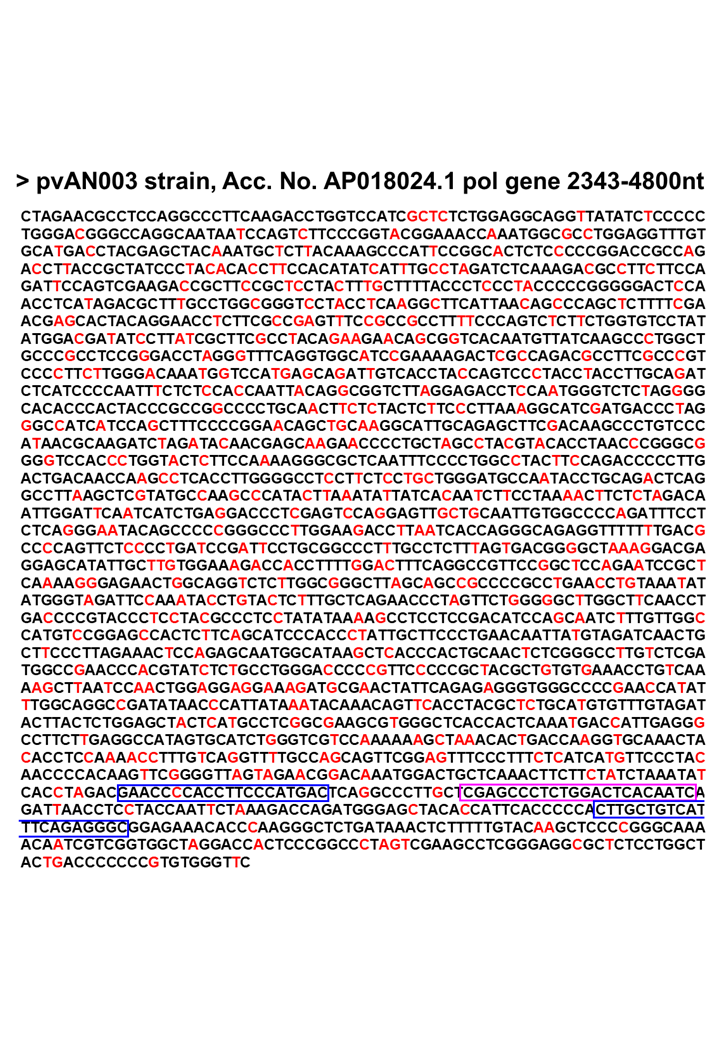

Supplement: FIG S3 [file msphere.00493-22-s0003.tif]
