# Supplementary material for: Identifying Pathogen and Allele Type Simultaneously in a Single Well Using Droplet Digital PCR
Source: mSphere. 2023 Jan 10;8(1):e00493-22. doi: 10.1128/msphere.00493-22 (PMC9942588; doi:10.1128/msphere.00493-22)
Supplement: TABLE S3 [file msphere.00493-22-s0006.docx]

**Table S3.** Genotyping of various alleles using IPATS-BLV

| Sample ID | Allele 1 | Allele 2 | *DRB3*009:02* ratio | *DRB3*016:01* ratio | The percentage of BLV-infected cells |
| --- | --- | --- | --- | --- | --- |
| #1 | **005:02* | **014:01:01* | 0.01191342 | 0.00036007 | ND^a^ |
| #2 | **010:01* | **015:01* | 0.01070361 | ND^a^ | ND^a^ |
| #3 | **001:01* | **011:01* | 0.02420252 | ND^a^ | ND^a^ |
| #4 | **006:01* | **010:01* | 0.00344443 | ND^a^ | ND^a^ |
| #5 | **014:01:01* | **027:03* | 0.00237900 | ND^a^ | 0.4758005 |
| #6 | **006:01* | **015:01* | 0.02617585 | ND^a^ | 49.794859 |
| #7 | **010:01* | **027:03* | 0.00487844 | 0.00112552 | 2.4779443 |
| #8 | **001:01* | **001:01* | 0.01094380 | ND^a^ | 15.252295 |
| #9 | **001:01* | **012:01* | 0.00580529 | 0.00041453 | 138.28632 |
| #10 | **010:01* | **012:01* | 0.00446447 | ND^a^ | 73.646647 |
| #11 | **015:01* | **027:03* | 0.00584656 | 0.00730837 | 103.99457 |
| #12 | **010:01* | **012:01* | ND^a^ | ND^a^ | 80.204463 |
| #13 | **001:01* | **027:03* | 0.00181486 | 0.00181486 | 4.7199491 |
| #14 | **001:01* | **010:01* | ND^a^ | ND^a^ | 11.779267 |
| #15 | **010:01* | **027:03* | 0.00612561 | ND^a^ | 37.153152 |
| #16 | **002:01* | **012:01* | 0.01164793 | 0.00582382 | 29.136074 |
| #17 | **001:01* | **027:03* | 0.00181654 | 0.00544986 | 79.942411 |
| #18 | **011:01* | **012:01* | 0.00429758 | ND^a^ | 74.969059 |
| #19 | **001:01* | **001:01* | ND^a^ | ND^a^ | 74.046493 |
| #20 | **001:01* | **012:01* | 0.00211347 | 0.00211347 | 98.168093 |
| #21 | **010:01* | **015:01* | 0.00956051 | ND^a^ | 41.497043 |
| #22 | **011:01* | **027:03* | 0.00670784 | 0.00335384 | 71.959674 |
| #23 | **011:01* | **015:01* | 0.00681452 | ND^a^ | 67.910761 |
| #24 | **010:01* | **027:03* | ND^a^ | ND^a^ | 88.39801 |
| #25 | **011:01* | **015:01* | 0.01892364 | 0.00630761 | 79.582584 |
| #26 | **012:01* | **027:03* | 0.00606147 | ND^a^ | 91.92639 |
| #27 | **010:01* | **015:01* | 0.00388764 | ND^a^ | 61.278611 |
| #28 | **010:01* | **011:01* | ND^a^ | ND^a^ | 98.273414 |
| #29 | **009:02* | **015:01* | 0.41412233 | ND^a^ | ND^a^ |
| #30 | **002:01* | **012:01* | 0.00918637 | ND^a^ | 0.166988431 |
| #31 | **002:01* | **012:01* | ND^a^ | ND^a^ | ND^a^ |
| #32 | **005:03* | **016:01* | 0.00368242 | 0.45545114 | 16.566636 |
| #33 | **002:01* | **012:01* | 0.00955920 | ND^a^ | 0.802813 |
| #34 | **002:01* | **012:01* | 0.00701826 | ND^a^ | 0.0519577 |
| #35 | **005:03* | **002:01* | 0.00838206 | 0.00038352 | 17.991567 |
| #36 | **005:02* | **012:01* | 0.00662005 | ND^a^ | 1.7654261 |
| #37 | **009:02* | **015:01* | 0.46336997 | ND^a^ | ND^a^ |
| #38 | **005:03* | **034:01* | 0.00431248 | 0.00177072 | ND^a^ |
| #39 | **009:02* | **015:01* | 0.48082397 | ND^a^ | ND^a^ |
| #40 | **009:02* | **015:01* | 0.50310229 | ND^a^ | ND^a^ |
| #41 | **007:01* | **009:02* | 0.48387558 | 0.00360128 | ND^a^ |
| #42 | **007:01* | **009:02* | 0.48879834 | 0.00161369 | ND^a^ |
| #43 | **009:02* | **016:01* | 0.46766509 | 0.47247611 | ND^a^ |
| #44 | **009:02* | **016:01* | 0.49878371 | 0.50402739 | ND^a^ |
| #45 | **009:02* | **016:01* | 0.49132784 | 0.46188088 | ND^a^ |
| #46 | **009:02* | **016:01* | 0.43059562 | 0.42381518 | ND^a^ |
| #47 | **009:02* | **016:01* | 0.42206102 | 0.43270428 | ND^a^ |
| #48 | **009:02* | **010:01* | 0.47509541 | 0.00031968 | ND^a^ |
| #49 | **009:02* | **010:01* | 0.42521606 | ND^a^ | ND^a^ |
| #50 | **001:01* | **018:01* | 0.00692286 | 0.00060168 | ND^a^ |
| #51 | **012:01* | **015:01* | ND^a^ | ND^a^ | ND^a^ |
| #52 | **044:01* | **045:01* | ND^a^ | ND^a^ | ND^a^ |
| #53 | **012:01* | **014:01:01* | 0.00821650 | 0.00027370 | ND^a^ |
| #54 | **008:01* | **045:01* | 0.00707893 | 0.00048788 | ND^a^ |
| #55 | **037:01* | **044:01* | 0.00829384 | 0.19204542 | 0.1381294 |
| #56 | **005:02* | **009:02* | 0.47631446 | 0.00004299 | ND^a^ |
| #57 | **018:01* | **028:01* | 0.00143383 | ND^a^ | ND^a^ |
| #58 | **015:01* | **016:01* | 0.00252116 | 0.50207961 | 101.9198895 |

^a^ No positive droplet was detected.
