# Supplementary material for: Identifying Pathogen and Allele Type Simultaneously in a Single Well Using Droplet Digital PCR
Source: mSphere. 2023 Jan 10;8(1):e00493-22. doi: 10.1128/msphere.00493-22 (PMC9942588; doi:10.1128/msphere.00493-22)
Supplement: TABLE S4 [file msphere.00493-22-s0007.docx]

**Table S4.** Sequences of tested alleles that have similar sequences with *DRB3*016:01* and **009:02* at IPATS-BLV primer/probe sites

|  | Sequence (5' to 3') | | |
| --- | --- | --- | --- |
|  | *DRB3*016:01* assay |  |  |
|  | Forward primer | Reverse primer | Probe |
| Primer/Probe | TTCGTGCGCTTCGAT | CGCTGCACAGTGAAACTCTCA | AGGACTTCCTGGAGGAGAA |
| *DRB3*071:01* | TTCGTGCGCTTCGAC | CGCTGCACAGTGAAACTCTCA | AGGACTTCCTGGAGGAGAA |
| *DRB3*100:01* | TTCGTGCGCTTCGAT | CGCTGCACAGTGAAACTCTCC | AGGACTTCCTGGAGGAGAA |
| *DRB3*100:05* | TTCGTGCGCTTCGAT | CGCTGCACAGTGAAACTCTCA | AGGACTTCCTGGAGCGGAA |
|  | *DRB3*009:02* assay |  |  |
|  | Forward primer | Reverse primer | Probe |
| Primer/Probe | GTGCGGTTCCTGGAG | CGCTGCACAGTGAAACTCTCA | AGATCCTGGAGGAGAGGC |
| *DRB3*009:01* | GTGCGGTTCCTGGAG | CGCTGCACAGTGAAACTCTCA | AGATCCTGGAGCGGAAGC |
| *DRB3*024:01* | GTGCGGTTCCTGGAC | CGCTGCACAGTGAAACTCTCA | AGATCCTGGAGGAGAGGC |

Deferent Nucleotides with primer/probe sequences are indicated by red color.
