# Supplementary material for: Identifying Pathogen and Allele Type Simultaneously in a Single Well Using Droplet Digital PCR
Source: mSphere. 2023 Jan 10;8(1):e00493-22. doi: 10.1128/msphere.00493-22 (PMC9942588; doi:10.1128/msphere.00493-22)
Supplement: TABLE S5 [file msphere.00493-22-s0008.docx]

**Table S5.** List of aligned BLV strains to determine conserved region

| Name of strain | Genotype | Acc. No. |  | Name of strain | Genotype | Acc. No. |
| --- | --- | --- | --- | --- | --- | --- |
| AB934282.1^a^ | 1 | AB934282.1 |  | pvAJ013 | 1 | AP019577.1 |
| JOTK | 1 | AB987702.1 |  | pvAJ014 | 1 | AP019578.1 |
| pvAF060 | 1 | AP018006.1 |  | pvAJ015 | 1 | AP019579.1 |
| pvAF076 | 1 | AP018007.1 |  | pvAJ016 | 1 | AP019580.1 |
| pvAF193 | 1 | AP018008.1 |  | pvAJ017 | 1 | AP019581.1 |
| pvAF245 | 1 | AP018009.1 |  | pvAJ018 | 1 | AP019582.1 |
| pvAF266 | 1 | AP018010.1 |  | pvAJ019 | 1 | AP019583.1 |
| pvAF293 | 1 | AP018011.1 |  | pvAJ021 | 1 | AP019585.1 |
| pvAF438 | 1 | AP018012.1 |  | pvAJ022 | 1 | AP019586.1 |
| pvAF481 | 1 | AP018013.1 |  | pvAJ023 | 1 | AP019587.1 |
| pvAF513 | 1 | AP018014.1 |  | pvAJ024 | 1 | AP019588.1 |
| pvAF746 | 1 | AP018015.1 |  | pvAJ025 | 1 | AP019589.1 |
| pvAF784 | 1 | AP018016.1 |  | pvAJ026 | 1 | AP019590.1 |
| pvAF805 | 1 | AP018017.1 |  | pvAJ027 | 1 | AP019591.1 |
| pvAF902 | 1 | AP018018.1 |  | pvAJ028 | 1 | AP019592.1 |
| pvAF982 | 1 | AP018019.1 |  | pvAJ029 | 1 | AP019593.1 |
| pvAK001 | 1 | AP018020.1 |  | pvAJ030 | 1 | AP019594.1 |
| pvAK006 | 1 | AP018021.1 |  | pvAJ031 | 1 | AP019595.1 |
| pvAK007 | 1 | AP018022.1 |  | pvAJ032 | 1 | AP019596.1 |
| pvAK011 | 1 | AP018023.1 |  | pvAJ033 | 1 | AP019597.1 |
| pvAN003 (Reference in this study) | 1 | AP018024.1 |  | pvAJ034 | 1 | AP019598.1 |
| pvAN004 | 1 | AP018025.1 |  | LS3 | 1 | HE967303.1 |
| pvAN006 | 1 | AP018026.1 |  | K02120.1^a^ | 1 | K02120.1 |
| pvAN008 | 1 | AP018027.1 |  | *469, deficient type | 1 | LC005616.1 |
| pvAN009 | 1 | AP018028.1 |  | par7 | 1 | LC080653.1 |
| pvAN011 | 1 | AP018029.1 |  | pvAF019 | 1 | LC164084.1 |
| pvAN013 | 1 | AP018030.1 |  | pvAF967 | 1 | LC164085.1 |
| pvAN014 | 1 | AP018031.1 |  | pvAN903 | 1 | LC164086.1 |
| pvAN015 | 1 | AP018032.1 |  | BLV_BL3.1 | 1 | LC436098.1 |
| pvAJ001 | 1 | AP019565.1 |  | IBK1705 | 1 | LC552969.1 |
| pvAJ002 | 1 | AP019566.1 |  | V50F13 | 1 | MH170027.1 |
| pvAJ003 | 1 | AP019567.1 |  | Arg41 | 2 | FJ914764.1 |
| pvAJ004 | 1 | AP019568.1 |  | AF033818.1^a^ | 4 | AF033818.1 |
| pvAJ005 | 1 | AP019569.1 |  | par62 | 6 | LC080656.1 |
| pvAJ006 | 1 | AP019570.1 |  | par91 | 6 | LC080658.1 |
| pvAJ007 | 1 | AP019571.1 |  | CHI-DQ | 6 | MG800834.1 |
| pvAJ008 | 1 | AP019572.1 |  | mon28 | 9 | LC080662.1 |
| pvAJ009 | 1 | AP019573.1 |  | por2 | 9 | LC080664.1 |
| pvAJ010 | 1 | AP019574.1 |  | por14 | 9 | LC080665.1 |
| pvAJ011 | 1 | AP019575.1 |  | por57 | 9 | LC080670.1 |
| pvAJ012 | 1 | AP019576.1 |  | QH2 | 10 | MF580995.1 |

^a^ If strain name is not specific, named by accession number.
